# Correction: Boroaluminosilicate geopolymers: role of NaOH concentration and curing temperature

**DOI:** 10.1039/d3ra90034g

**Published:** 2023-04-26

**Authors:** Ali Nazari, Ali Maghsoodpoor, Jay G. Sanjayan

**Affiliations:** a Centre for Sustainable Infrastructure, Faculty of Science, Engineering and Technology, Swinburne University of Technology Victoria 3122 Australia alinazari@swin.edu.au +61 3 92148370; b Department of Geopolymer and Concrete, WorldTech Scientific Research Center (WT-SRC) Tehran Iran

## Abstract

Correction for ‘Boroaluminosilicate geopolymers: role of NaOH concentration and curing temperature’ by Ali Nazari *et al.*, *RSC Adv.*, 2015, **5**, 11973–11979, https://doi.org/10.1039/C4RA11665H.

The authors regret that the name of one of the authors (Ali Maghsoodpoor) was shown incorrectly in the original article. The corrected author list is as shown above.

The Royal Society of Chemistry apologises for these errors and any consequent inconvenience to authors and readers.

## Supplementary Material

